# A Comparison of Traditional and Novel Methods for the Separation of Exosomes from Human Samples

**DOI:** 10.1155/2018/3634563

**Published:** 2018-07-26

**Authors:** Li-Li Yu, Jing Zhu, Jin-Xia Liu, Feng Jiang, Wen-Kai Ni, Li-Shuai Qu, Run-Zhou Ni, Cui-Hua Lu, Ming-Bing Xiao

**Affiliations:** ^1^Department of Gastroenterology, Affiliated Hospital of Nantong University, Nantong, Jiangsu 226001, China; ^2^Research Center of Clinical Medicine, Affiliated Hospital of Nantong University, Nantong, Jiangsu 226001, China

## Abstract

Exosomes are discrete populations of small (40-200 nm in diameter) membranous vesicles that are released into the extracellular space by most cell types, eventually accumulating in the circulation. As molecular messengers, exosomes exert a broad array of vital physiologic functions by transporting information between different cell types. Because of these functional properties, they may have potential as biomarker sources for prognostic and diagnostic disease. Recent research has found that exosomes have potential to be utilized as drug delivery agents for therapeutic targets. However, basic researches on exosomes and researches on their therapeutic potential both require the existence of effective and rapid methods for their separation from human samples. In the current absence of a standardized method, there are several methods available for the separation of exosomes, but very few studies have previously compared the efficiency and suitability of these different methods. This review summarized and compared the available traditional and novel methods for the extraction of exosomes from human samples and considered their advantages and disadvantages for use in clinical laboratories and point-of-care settings.

## 1. Introduction

Exosomes, which were originally described as small vesicles with a diameter of 40-200 nm secreted by reticulocytes (immature erythrocytes), are lipid-bilayer-enclosed extracellular vesicles containing proteins and nucleic acids (RNA), but without organelles [1]. Unlike extranuclear granules and apoptotic bodies, exosomes are of endocytic origin, from inward budding of the endosomal compartment within a cell, forming a multivesicular body which subsequently fuses with the plasma membrane for release. Furthermore, exosomes contain both their own unique biomarkers, such as CD9 and CD81, and associated proteins and genetic materials (microRNAs, IncRNAs, circleRNAs, etc.) of their precursor cells. The role of exosomes in cell-cell communication, disease diagnosis, and drug delivery and as a possible source of biomarkers has attracted great interest among researchers, leading to a surge in exosome research.

Recent studies have reported that exosomes can be secreted by many types of cells and can also be isolated from a range of body fluids, including plasma, bile, urine, breast milk, saliva, pleural fluid, ascites, and bronchoalveolar lavage fluid [2]. Therefore, exosomes have a wide range of sample types and are readily available. And long-term storage at −80°C does not affect exosome properties. In addition, the presence of exosomes in urine and saliva is expected to replace the traditional invasive body fluid collection and achieve the purpose of clinically noninvasive diagnosis. Exosomes are thought to be associated with intercellular communication, by facilitating the exchange of proteins and lipids between the exosome-producing cells and target cells [3], and through the horizontal transfer of biomolecular substances between cells and their microenvironment, as well as through regulating the expression of receptor cells and the activation of signaling pathways [4].

As an important carrier for cell signaling molecules (proteins and nucleic acids), exosomes are known to actively take part in tumor initiation, progression, and metastasis, via altering the tumor microenvironment [5]. Exosomes have also been shown to be abundant in complex biological fluids, especially in peripheral blood, which plays an important role in a variety of pathophysiological processes. Further research has implicated tumor-derived exosomes as being involved in cancer progression and metastasis [6]. Against this background, exosomes are considered to be one of the most promising breakthrough directions for cancer research in the next decade.

Recently, a number of studies have demonstrated important physiological roles of exosomes in the immune, cardiovascular, and nervous systems, as well as in the pathogenesis of a range of diseases including cancer [7]. Thus, through further research on exosomes it may be possible to gain a deeper understanding of the molecular mechanisms of such diseases. Exosomes released from tumor cells have recently received considerable attention because they have been shown to contain biomarkers such as tumor-specific proteins and nucleic acids that are indicative of a cancer's stage and progression. On this basis, exosomes in body fluids have emerged as a promising source of cancer biomarkers for potential use in diagnosis, prognostication, and treatment monitoring [8]. In addition, exosomes are increasingly being seen as possible alternatives to liposomes as drug delivery vehicles for tumor immunotherapy without inducing a host immune response [9], which further highlights the clinical potential of exosomes. Most of the potential targets for tumor therapy are cancer specific biomarkers. Therefore, it is of great potential significance to study on the tumor biomarkers that may be present on exosomes, in order to inform the development of future tumor-targeting therapies.

Despite there is a current lack of technical standardized tools or methods by which to consistently isolate high-yield and high-purity intact exosomes, this poses a major current challenge to current research on exosomes for the discovery of reliable biomarkers of disease [8]. Such methods are needed in order to produce high-purity, loss-free exosome samples that can be used as a basis for further exploration of their functional mechanisms and composition.

The purpose of this review is to consider and compare the various methods available for the purification and characterization of exosomes. Before performing any functional analysis, it is critical to ensure that the purified vesicles are exosomes with no contaminating materials. The first part described the traditional methods that can be used to separate exosomes from samples, and the second part described novel methods for characterizing and assessing the purity of the isolated exosomes [3].

## 2. Traditional Methods for the Extraction of Exosomes from Human Samples

Traditional methods for the isolation of exosomes from human samples include ultracentrifugation, filtration, immunoaffinity isolation, polymeric precipitation isolation, and liquid chromatography techniques [10].

### 2.1. Differential Centrifugation/Ultracentrifugation

**Figure 1:**
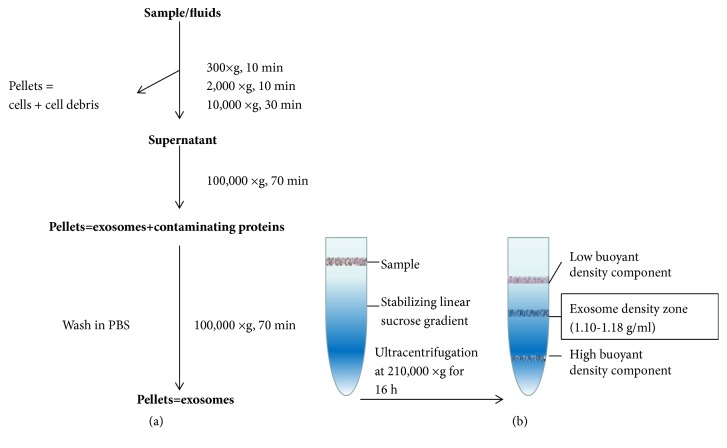
Flow chart of the procedure for the purification of exosomes from human samples by ultracentrifugation and density gradient centrifugation. (a) After a number of differential centrifugations at low speed, the pellets (cells, cell debris) are discarded, and the supernatant is kept for the next centrifugation step. In contrast, after the two 100,000×g centrifugations, the pellets (containing exosomes with contaminant proteins as well as pure exosomes) are kept, and the supernatants are discarded. (b) The sample is added to an inert gradient medium for ultracentrifugation. Different components of the sample settle to their isodensity areas, allowing separation of the exosome and other components in the sample.

The most widely applied and most basic method for the isolation of exosomes from all kinds of human samples is differential centrifugation [11, 12]. The principle of this method is to separate out the exosomes from the other materials present in the sample based on their volume and physical properties. In brief, the collected samples are centrifuged at different speeds to remove cells, cellular debris, and macromolecular proteins, followed by ultracentrifugation at 100,000×g for 70 minutes to obtain the exosomes in the supernatant (Figure 1(a)). All of the centrifugation steps are carried out at 4°C [13].

Although differential centrifugation/ultracentrifugation is effective for the isolation of exosomes, the technique is time-consuming, labor-intensive, and heavily instrument-dependent for both research laboratories and clinical settings alike [14, 15]. In addition, the ultracentrifugation process may result in a large number of exosomes being lost, at the risk of reducing the yield [10]. Therefore, ultracentrifugation is considered inappropriate for the extraction of exosomes from a small amount of serum samples and results in lack of purity. With differential centrifugation, successive rounds of centrifugation are intended to pellet consecutively the apoptotic bodies, cell debris, and shedding vesicles as well as the exosomes [11]. Electron microscopy identifies the exosomes as having gathered into clumps during this process. However, due to overlaps in the vesicle sizes of exosomes, microbubbles, and apoptotic bodies, the isolation of pure exosomes from microbubbles and apoptotic bodies via differential centrifugation is impossible [16]. Since there are no uniform standards for the identification of microbubbles and exosomes, this has resulted in some studies having suggested that the final centrifugation product is microbubbles rather than exosomes. On the other hand, the proteins and RNA components that are present on the exosomes are not greatly affected during ultracentrifugation, and it is considered suitable for larger samples such as cell supernatant and urine, and for protein research. Nevertheless, because of the cumbersome nature of the ultracentrifugation process and the dependence of the method on the equipment, the generality of the method is limited.

### 2.2. Density Gradient Centrifugation

In density gradient zone centrifugation (hereinafter referred to as zone centrifugation), the sample is added to an inert gradient medium for centrifugal sedimentation or sedimentation equilibration. The basis of the method is that, under a certain centrifugal force, the different components of the sample will settle to their isodensity zone, thus achieving the separation of exosomes from other components in the sample. The most commonly used of these methods is the sucrose density gradient centrifugation method. A linear sucrose gradient (2.0-0.25 M sucrose) is built into an ultracentrifuge tube, and the samples are then deposited at the top of this linear sucrose gradient. The gradients are then ultracentrifuged for 16 h at 210,000×g at 4°C [17]. This method yields single exosomes banding at their characteristic density zone (1.10-1.18 g/ml) (Figure 1(b)) [18].

Compared with traditional ultracentrifugation, the advantages of the method are, firstly, that it has higher separation efficiency, thus resulting in exosomes of greater purity. Secondly, the exosome particles are unlikely to be crushed or deformed during the separation process, and the separated components are prevented from mixing again. The shortcomings of the method are that the centrifugation takes longer time, and the end-product yield is not as high. Furthermore, it is necessary to prepare an inert gradient media solution in advance of running the method, and both the preparatory work and method itself are complex and time-consuming to perform. The instruments required for density gradient centrifugation are also expensive and take up significant space in the laboratory, which prevents many laboratories from acquiring them [16].

Additional research on the use of density gradient centrifugation for the separation of exosomes has shown that use of a 5-40% iodixanol gradient instead of sucrose improves the separation of exosomes from viral particles and small apoptotic bodies. Moreover, unlike sucrose, iodixanol is capable of forming isoosmotic solutions at all densities and is thus better at preserving the size of the vesicles during their passage through the gradient [19, 20]. A further advantage of the density gradient method is that it is not prone to capture contaminating cellular debris within the separation process, but it is also highly user intensive and not suitable for high-throughput applications [21].

### 2.3. Extraction Using Immunomagnetic Beads

Immunomagnetic beads are spherical magnetic particles that are coated with a monoclonal antibody that specifically binds to a target substance. Immunomagnetic extraction of exosomes is based on the specific binding between these antibodies and certain receptor molecules present on the surface of the exosomes. Membrane surface receptor molecules such as CD9, CD63, and CD81 are used to extract the exosomes using immunoaffinity capture methods [22, 23].

According to the method, the immunomagnetic beads are first coated with antibodies against the exosome-associated receptor molecules, as described above, and these are incubated with the sample to form exosome-magnetic bead complexes. Next, under the attraction of an applied magnetic field, directional movement of these complexes can be induced for the purpose of separating the exosomes from the sample. The advantage of this approach is that it is target specific and ensures the integrity of the extracted exosomes. The method is also relatively easy to carry out and does not require expensive instrumentation. In addition, it allows for the selection and extraction of a specific exosome subpopulation from the sample, based on the expression of specific markers, irrespective of vesicle size. It is well known that different types of cells may generate different sizes of exosome, but all exosomes carry the same surface markers [24]. However, the method is not without its challenges. It is quite difficult to elute the exosomes from the magnetic beads, which can present a major limitation since the bound exosomes cannot then be used in downstream experiments [13]. Immunoisolation-based devices, albeit conferring a shorter assay time than other methods (of around 1.5 h), require the use of sophisticated analytical tools to analyze exosomes when extracted from plasma, which does not make the method practical or user-friendly for point-of-care testing [14]. Thus, the method cannot be applied to all sample types. Furthermore, the reagents are expensive and the nonneutral pH and nonphysiological salt concentrations that are employed are likely to affect exosome biological activity, limiting the possibilities for further experimentation on the extracted exosomes.

### 2.4. Extraction Using Exoquick™: Exosome Precipitation Reagent

Exoquick™ is a commercially available exosome precipitation reagent that is now commonly used to extract exosomes from human samples. It is based upon the principle of compound polymerization precipitation. Samples are mixed with the Exoquick™reagent as described in the manufacturer's instructions and react with each other to form a mesh-like polymeric web that captures exosomes of a certain size, usually between 60 and 180 nm in diameter. These are later pelleted at low centrifugal speed. The method is simple and fast and requires only a basic centrifuge [25]. Furthermore, exosomes extracted by this method are highly uniform in their size and the method is suitable for the extraction of exosomes from small samples, such as serum samples. At the same time, the method effectively excludes macromolecular proteins that may be present in samples from being extracted, which facilitates subsequent analysis of the extracted exosomes. However, the disadvantages are that certain contaminants, such as lipoproteins, may be coextracted with the exosomes, which impair their subsequent analysis. Moreover, since polymeric precipitation extracts exosomes of 60-180 nm in diameter, the method cannot be used for the extraction and assessment of larger exosomes that may be present in a sample [13]. In addition, Exoquick™ is expensive, and a single reagent dose only allows the analysis of a small number of samples, which can put a significant financial burden on clinics where the throughput of samples may be high.

### 2.5. Chromatography

Chromatography is used to separate solutes based on the relative pore size of the chromatography gel relative to the size of the molecules in the sample and is generally coupled with a low-speed centrifugation step. The principle behind the technique is that particles in samples will move through the filtration column at different rates depending on their size [13]. Macromolecules cannot enter the gel pores and will thus be eluted by the first mobile phase that is passed through the column along the gaps between the porous gel particles. Smaller molecules will enter the gel pores and thus be retained in the column and eluted more slowly, to produce an eluent containing 40-200 nm diameter exosomes. The exosomes obtained by chromatography are typically of high purity and uniform in size when viewed under an electron microscope. However, the low extraction volume and extensive laboratory equipment requirements make the method difficult to apply widely in clinical or laboratory settings.

## 3. Novel Methods of Extraction of Exosomes

Because of the aforementioned limitations of traditional separation methods for the extraction of exosomes from human samples, many novel methods have been developed in recent years.

### 3.1. Ultrafiltration Separation Technology

Stirred ultrafiltration [26] can be used for the separation of exosomes from samples, based on the principle that the pore size of the ultrafiltration membrane allows substances of specific relative molecular mass to pass through or be intercepted. Solvent and small molecules will be filtered through the membrane, while the molecules with higher relative molecular masses will be trapped in the ultrafiltration membrane, thereby achieving separation. The use of this method for the separation of exosomes applies the same principles as centrifugal ultrafiltration, with the difference being that the latter relies on centrifugal force, rather than a membrane, to separate out the solvent and small molecules, which requires centrifugation at 100,000×g for 1-2 h to obtain exosomes. In stirred ultrafiltration, the pressure generated by the externally supplied nitrogen causes the sample to be passed through the ultrafiltration membrane to extract the exosomes.

Compared to traditional ultracentrifugation technology, the advantage of stirred ultrafiltration is that it is less time-consuming and avoids the need for an ultracentrifuge. In addition, stirred ultrafiltration results in the extraction of a greater number of exosomes per sample volume. Similar to ultracentrifugation, stirred ultrafiltration is also suitable for the extraction of exosomes from large samples including culture supernatant. Furthermore, ultracentrifugation requires a centrifugal force of 100,000×g, which generates tremendous pressure and can damage the integrity of the exosomes, whereas stirred ultrafiltration requires a pressure of only 517.125 kPa. This greatly reduces the likelihood of rupture of the exosomes, which may be one of the reasons why the method results in the extraction of a greater number of exosomes per sample volume. Finally, the end products obtained by stirred ultrafiltration do not become aggregated together, which makes it easier to use them for further studies.

Based on the above, it is suggested that stirred ultrafiltration can be used for the separation of exosomes based on its convenience, speed, efficiency, and affordability of equipment. However, there is the same concern regarding a lack of purity of the end-product as there is with ultrafiltration. It is difficult to exclude those compounds or molecules with a similar diameter to exosomes from being coextracted, such as microbubbles and apoptotic bodies.

### 3.2. Integrated Double Filtration Microfluidic Device

**Figure 2:**
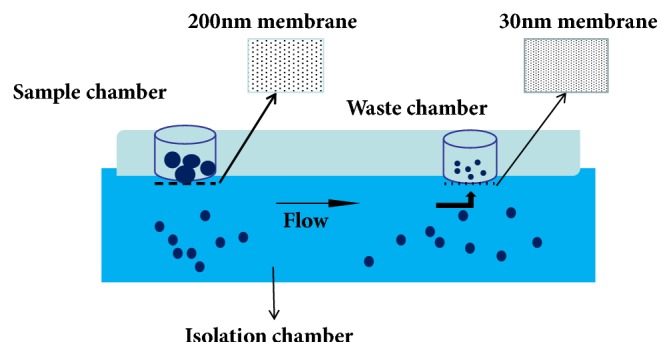
Schematic of an integrated double filtration microfluidic device for separation exosomes. Based on size-exclusion, particles larger than 200 nm are excluded by the membrane with a pore size of 200 nm in the sample chamber, whereas those smaller than 30 nm pass through the device into the waste chamber. Exosomes with a size between 30 and 200 nm are separated and enriched in the isolation chamber.

An integrated double filtration microfluidic device can be used for the separation of exosomes from samples. The device is built mainly on the principles of chromatography. The double filtration of the microfluidic device works through the presence of two membranes with pore sizes of 200 and 30 nm in diameter. According to the principles of size-exclusion chromatography, particles that are larger than 200 nm are excluded by the membrane with a pore size of 200 nm in the sample chamber, and similarly particles smaller than 30 nm pass through this membrane and into the waste chamber. Particles of a size that is between these outer boundaries remain in the sample chamber, thus achieving the extraction of exosomes (Figure 2). The device enriches exosomes much more efficiently than ultracentrifugation, which is costly and time-consuming. Moreover, because the double filtration device is built on the principles of size-exclusion, it eliminates the need for a capture antibody to be used to isolate the exosomes. Besides, the integrated, inexpensive, and disposable nature of the microfluidic device makes it more suitable than other analytical methods for the extraction of exosomes in point-of-care testing. The disadvantages of the method are that during the double filtration process exosomes may be squeezed and broken into undetectable particles [14], which can affect the extraction yield of exosomes. Like the ultrafiltration and ultracentrifugation methods, the integrated double filtration microfluidic device has the same disadvantage regarding inefficiency of purity of the extracted product.

### 3.3. Nanoplasmon-Enhanced Scattering (nPES)

**Figure 3:**
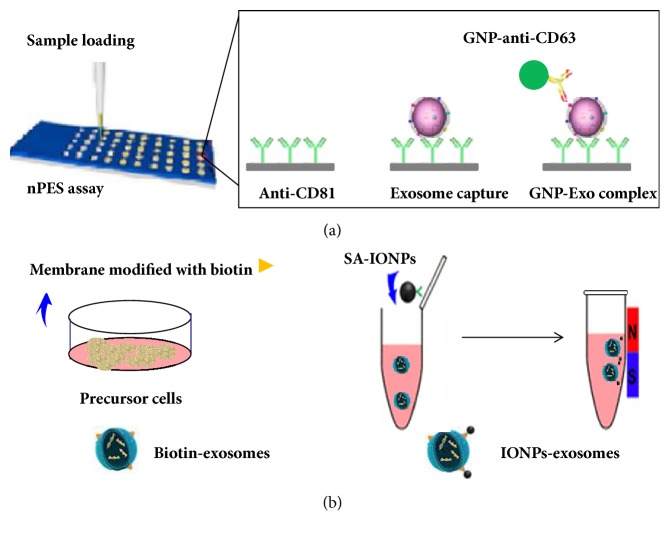
The scheme for the nPES exosomes enrichment and for membrane-mediated exosomes separation. (a) The anti-CD81 antibody is conjugated to the sensor chip surface to enrich the exosomes in the sample. Subsequently, it reacts with antibody-coated gold nanoparticle probes (GNPs) to form GNPs-exosome complexes. (b) When donor cells are modified with biotin, exosomes of origin can be specifically bound to magnetic nanoparticle-exosome complexes with streptavidin-coupled magnetic nanoparticle (SA-IONPs). The complexed-exosomes are separated and enriched from the sample with a magnetic field.

The principle of nPES-enriched exosomes is similar to that of enzyme-linked immunosorbent assay (ELISA). The method uses antibodies against the cellular markers CD81, CD63, and CD9, which are enriched on most exosome membranes, to both capture and detect all exosomes present in a sample. The steps are as follows, firstly, the sensor chip is prepared for sample application. An anti-CD81 antibody is conjugated to the silica surface of a sensor chip, so that all exosomes that express this common exosome marker are captured and enriched when the wells of this chip are loaded with samples containing exosomes from any cell type. Subsequently, antibody-coated gold nanoparticle probes (GNPs) are used to react with the sensor chip, so that binding of GNP with immobilized exosomes on the chip surface forms GNPs-exosome complexes (Figure 3(a)). This is why exosomes can be separated from the sample. Then under dark-field microscopy, different GNPs scatter different colors to reflect the amount of exosomes contained. The nPES platform can be used as a rapid, high-throughput, sensitive, and specific method for the detection of exosomes from trace samples [9]. In addition, depending on the amount of scatter area, the extracted exosomes can be quantitatively analyzed based on calculation of the proportion of the area that contains scattered light. However, the acquisition of specific antibodies for the detection of exosome proteins can be costly. In addition, complex statistical tools are required to detect the amount of radiation under dark-field microscopy [5].

### 3.4. Membrane-Mediated Exosome Separation

This method is based on an improvement of the method that uses immunomagnetic beads for the extraction of exosomes. Thus, the principles of the two methods are analogous. Based on a donor cell-assisted membrane modification strategy, exosomes from precursor cells, modified with biotin, react with the magnetic nanoparticles to form magnetic nanoparticle-exosome complexes. In the presence of a magnetic field, the complexed-exosomes are separated and enriched from the sample (Figure 3(b)).

Compared with extraction using immunomagnetic beads, the biotin bound to exosomes corresponds to membrane surface molecular markers in membrane-mediated exosome separation, thereby avoiding the use of related antibodies. In addition, because the magnetic nanospheres are conjugated onto the surface of the exosomes, the nanoparticle-exosome complexes are easily separated from the samples in the presence of an external magnetic field. And this process itself does not destroy the exosomes and their protein and RNA contents. Furthermore, the moderate operating conditions of the method may better preserve the original structure of the exosomes by preventing aggregation and rupture. Moreover, the method is relatively simple and convenient, consisting of only one step and taking less than 2 h to complete. Finally, with the assistance of a magnetic field, exosomes that have been modified with magnetic nanoparticles demonstrated excellent tumor-targeting activity. For example, these exosomes can be transformed into antitumor delivery platforms by directly loading doxorubicin via electroporation. When applying a magnetic field to the tumor site, the engineered exosomes loaded with antitumor drugs can exhibit significant tumor growth inhibition effects [16], which will significantly promote the development of natural therapeutic nanoplatforms.

Following on from the above, based on the modification of precursor cell membranes, cell-released exosomes can then easily be purified and enriched by membrane-mediated exosome separation, a convenient procedure with good specificity, high efficiency, and reliable reproducibility, making it possible for exosomes to be utilized as drug carriers for the treatment of tumors. However, the method still has some limitations that restrict its application in the laboratory. For example, the process of achieving binding of precursor cells and biotin is complicated. And the influence of microbubbles on this process cannot be ruled out, so the purity of the extracted exosomes cannot be guaranteed [16].

### 3.5. On-Chip Isolation of Exosomes

**Figure 4:**
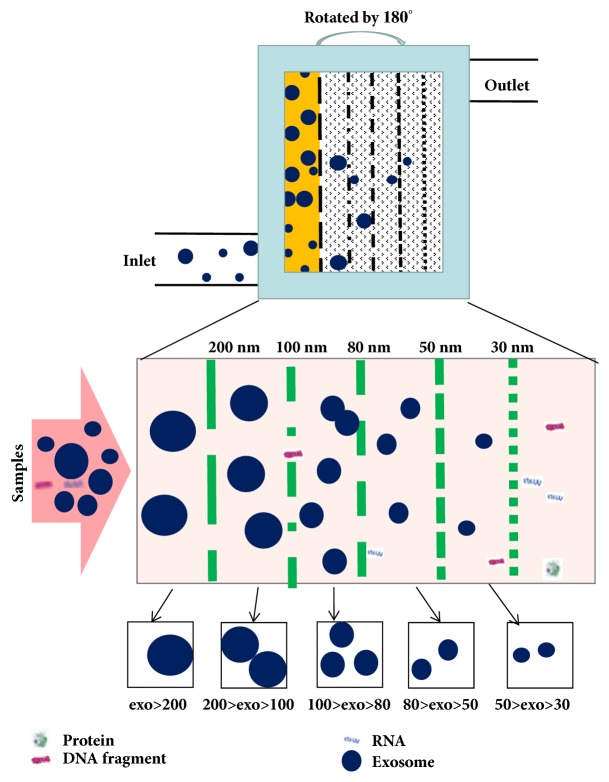
Schematic illustration of the ExoTIC device for exosomes isolation. Samples enter at a certain flow rate from the inlet, then the device rotates repeatedly. The sample is enriched in the complete exosomes in the size range of 30-200 nm through the nanoporous membrane. Free nucleic acids, proteins, lipids, and other small fragments were flushed out and concentrated exosomes were collected from a filter membrane using a standard pipette.

Some researchers have utilized a lab-on-a-chip device for the extraction of exosomes: the exosome total isolation chip (ExoTIC) [8]. The ExoTIC has been specifically designed to simplify the extraction of exosomes. It uses a simple filtration approach in which exosomes contained in clinical samples, including plasma, culture media, urine, and lavage, are passed through a nanoporous membrane to enrich and purify intact exosomes in the 30-200 nm size range. Free nucleic acids, proteins, lipids, and other small fragments are flushed out, and the concentrated exosomes are collected from the filter membrane using a standard pipette. The protocol for isolating the exosomes from samples is as follows: a defined amount of the sample solution is introduced continuously into the ExoTIC device via a syringe, using a syringe pump at a constant flow rate of 5 ml/h. Once the sample has been concentrated down, the ExoTIC was rotated by 180° so that the exosomes were enriched on the corresponding filtration membrane, while small proteins and free nucleic acids would be flushed out of the outlet (Figure 4). The ExoTIC technology presents several advantages over traditional methods for exosome isolation. Because it is simple, fast, cost-effective, and scalable and produces a high-yield of exosomes from clinical samples, the technology can be applied to point-of-care testing of exosomes as part of disease diagnostics and can be used in resource limited settings. Furthermore, because the method achieves a high-yield of extracted exosomes even from small sample sizes, the extracted product can be used in a large number of further applications or studies.

## 4. Future Outlook

It is well established that various human cell types can release exosomes, which carry membrane-tethered as well as intravesicular molecules to distant cellular targets [18]. In recent years, exosomes have become a research focus because of their unique functions in cell behavior and their potential application to disease diagnosis [27]. With further research, it was discovered that exosomes could be used not only to understand disease progression and as biomarkers, but also as potential carriers for drug therapy. Compared with traditional peripheral blood tumor markers, certain exosome-derived proteins and miRNAs have a higher diagnostic value for tumors. For example, it is well known that pancreatic cancer is a malignant tumor with a poor prognosis. Carbohydrate antigen 19-9 (CA19-9) is a pancreatic cancer tumor marker widely used clinically. Unfortunately, the false positive rate of early diagnosis of this indicator is very high and cannot be routinely used as an oncologic marker for the diagnosis of early pancreatic cancer. However, it has been found that glypican-1 (GPC1), a cell surface proteoglycan, specifically enriched on pancreatic cancer-cell-derived exosomes, is of significance for the diagnosis of early pancreatic cancer [1]. In conclusion, exosomes may become an important part of the clinical tools available for the diagnosis and treatment of diseases.

In recent years, the possible use of exosomes in cancer therapy has become a research hotspot, and having accurate, reliable, and accessible methods for the extraction of exosomes from samples is fundamental to that research [28]. However, the current lack of a standardized approach to the separation and characterization of exosomes in human samples may potentially hinder our ability to investigate the potential applications for exosomes in the diagnosis and treatment of disease. Therefore, there is a strong need to establish a standardized method by which to achieve the extraction of exosomes in high concentration and with a high level of purity [29].

Currently, a variety of techniques exist for the extraction of exosomes from human samples. The most common of these include traditional ultracentrifugation, density gradient separation, immunomagnetic beads, and ultrafiltration. However, these methods always have various defects in many aspects such as equipment requirements, expensive reagents, operating procedures, productivity, and purity (Table 1). And in recent years, there have also been a number of novel methods developed, such as microfluidic devices, nPES, membrane-mediated exosome separation, and ExoTIC. However, because they are currently limited to the theoretical stage and lack of preclinical research and experience, therefore their stretchability and reliability are unknown, so not yet been developed and applied [30].

There is currently no a specific exosome separation technique suitable for every study or has been widely accepted. Some experts have proposed a combination of several separation techniques, but no consensus has yet been reached. Each exosome isolation technique has its own properties, the combination of which could increase the purity of the product. However, the challenges that must be overcome by integrating exosome separation techniques are higher costs, more sophisticated operations, and more separation steps. Based on the clinical application requirements for the diagnosis and treatment of diseases, exosomes have become a hot topic of clinical research because of their extensiveness, simplicity, and noninvasiveness. For example, exosomes can be extracted from all body fluids. The presence of exosomes in readily available body fluids such as urine and saliva may pave the way for future clinically noninvasive models. Furthermore, the isolation and analysis of exosomes are expected to replace traditional invasive collection way of body fluid such as blood, pleural fluid, ascites, and bronchoalveolar lavage fluid and cerebrospinal fluid, etc. [30].

Ideally, a standardized method for the separation of exosomes from human samples will be developed in the near future, which would facilitate the development of novel applications for exosomes in clinical laboratories.

## Figures and Tables

**Table 1:** The characteristics of each method.

		Equipment	Features	
			advantages	disadvantages
**Traditional Methods**	**Ultracentrifugation**	Ultracentrifuge	High sample capacity; Protein and RNA components are not affected; facilitating later research	Time-consuming; instrument-dependent; low purity
	**Density gradient**	Ultracentrifuge	High separation efficiency; high purity; won't to be crushed or deformed	Long run time; equipment dependence; low yield; complex process
	**Immunomagnetic Beads**	Magnetic bead, antibody	Save time; Maintain integrity; Convenient operation; Not affected by exosome size; No need for expensive instruments	High reagent cost; low capacity and low yields
	**Exoquick**™	Exoquick™	Simple steps, quick operation; size uniformity; suitable for small samples, such as serum	Impurity; Affected by exosome diameter; expensive reagents; low production
	**Chromatography**	Gel filtration column	High purity; uniform in size	Low extraction volume; extensive laboratory equipment requirements

**Novel Methods**	**Stirred ultrafiltration**	Ultrafiltration membrane, nitrogen	Do not rely on equipment; less time consuming; Reduces the destruction of exosomes during the process	Moderate purity of isolated exosomes; loss of exosomes during the process
	**Filtration Device**	Microfluidic device	Fast, low cost; easy automation and integration; high portability	Lack of standardization and large scale tests on clinical samples, lack of method validation; low sample capacity
	**nPES**	GNPs, antibodies	Fast, efficient, high purity; quantitative analysis	High reagent cost; complex statistical tools; low capacity
	**Membrane modification**	Magnetic field, magnetic nanoparticles	Need not antibodies; save time; preserve the original structure of the exosomes; drug carriers	Complicated operation; impurity
	**ExoTIC**	ExoTIC, syringe pump	Simple operation, exosome in a specific range, high purity	Special equipment requirements; Lack of tests on clinical samples,
